# Supplementary material for: Terminal type-specific cannabinoid CB1 receptor alterations in patients with schizophrenia: a pilot study
Source: bioRxiv. 2023 Apr 11:2023.04.11.536217. Preprint. [Version 1] doi: 10.1101/2023.04.11.536217 (PMC10120624; doi:10.1101/2023.04.11.536217)

bioRxiv preprint doi: https://doi.org/10.1101/2023.04.11.536217; this version posted April 11, 2023. The copyright holder for this preprint (which was not certified by peer review) is the author/funder, who has granted bioRxiv a license to display the preprint in perpetuity. It is made available under aCC-BY 4.0 International license.

Supplemental information

Table S1. Demographic, postmortem, and clinical characteristics of individual human subjects included in the study.

Table S2. Statistical results of sum CB1R intensity and mean CB1R intensity analyses without outlier pair.

Table S3. Mean values of Ctrl/SZ subject pair sum CB1R intensity ratios for pairs with and without cannabis and medication exposure history. Values are represented as mean ± SEM.

Table S4. Mean values and T-tests results of Ctrl/SZ subject pair mean CB1R intensity ratios for pairs with and without cannabis and medication exposure history for each terminal type. Values are represented as mean ± SEM.

Figure S1. Mean CB1R intensity frequency histograms for inhibitory (vGAT-IR) boutons from postmortem PFC samples of subjects with schizophrenia (SZ) and unaffected comparisons (Ctrl), measured in analogue-to-digital units (ADU). The line at 712 ADU denotes the median value of mean CB1R intensity for vGAT-IR boutons in Ctrl.

Figure S2. Mean CB1R intensity in excitatory (vGlut1-IR) boutons (top), low-CB1R-expressing inhibitory (vGAT-IR) boutons (middle), and high-CB1R-expressing inhibitory (vGAT-IR) boutons (bottom) for individual subjects across cortical layers. Each individual data point represents the mean intensity averaged across all sampled sites for a single subject. Central line indicates the median, box boundaries extend from the 25th to 75th percentiles, and whiskers

bioRxiv preprint doi: https://doi.org/10.1101/2023.04.11.536217; this version posted April 11, 2023. The copyright holder for this preprint (which was not certified by peer review) is the author/funder, who has granted bioRxiv a license to display the preprint in perpetuity. It is made available under aCC-BY 4.0 International license.

extend from the minimum to maximum value. There was a main effect of subject group for excitatory and high-CB1R-expressing inhibitory boutons, $p<0.001$. **$p<0.001$.

bioRxiv preprint doi: https://doi.org/10.1101/2023.04.11.536217; this version posted April 11, 2023. The copyright holder for this preprint (which was not certified by peer review) is the author/funder, who has granted bioRxiv a license to display the preprint in perpetuity. It is made available under aCC-BY 4.0 International license.

Table S1.

Healthy Comparison Subjects								Subjects with Schizophrenia											
Pair	Case	Sex/ Race	Age	PMI[a]	Storage Time[b]	pH	Cause of Death	Case	DSM IV diagnosis	Sex/ Race	Age	PMI[a]	Storage Time[b]	pH	Cause of Death	History of Cannabis Use	Antipsychotic ATOD	Antidepressant ATOD	Benzodiazepine / VPA ATOD
1	681	M/W	51	11.6	190	7.2	Hypertrophic cardio-myopathy	640	Chronic paranoid schizophrenia	M/W	49	5.2	195	6.9	Pulmonary embolism	None	Y	Y	N
2	685	M/W	56	14.5	189	6.6	Hypoplastic coronary artery	622	Chronic undifferentiated schizophrenia	M/W	58	18.9	197	6.8	Right MCA infarction	None	N	N	N
3	592	M/B	41	22.1	202	6.7	ASCVD	533	Chronic undifferentiated schizophrenia	M/W	40	29.1	212	6.8	Accidental Asphyxiation	None	Y	N	N
4	727	M/B	19	7.0	183	7.2	Trauma	829	Schizoaffective disorder; ADC; OAR	M/W	25	5.0	164	6.8	Suicide by salicylate overdose	Abuse in remission	N	N	Y
5	700	M/W	42	26.1	187	7.0	ASCVD	539	Schizoaffective disorder; ADR	M/W	50	40.5	211	7.1	Suicide by combined drug overdose	None	Y	Y	Y
6	551	M/W	61	16.4	209	6.6	Cardiac tamponade	625	Chronic disorganized schizophrenia; AAC	M/B	49	23.5	197	7.3	ASCVD	None	Y	Y	N
7	604	M/W	39	19.3	200	7.1	Hypoplastic coronary artery	581	Chronic paranoid schizophrenia; ADC; OAC	M/W	46	28.1	204	7.2	Accidental combined drug overdose	Use	Y	N	Y
8	818	F/W	67	24.0	167	7.1	Anaphylactic reaction	917	Chronic undifferentiated schizophrenia	F/W	71	23.8	147	6.8	ASCVD	None	Y	N	N
9	871	M/W	28	16.5	155	7.1	Trauma	878	Disorganized schizophrenia; ADC	M/W	33	10.8	154	6.7	Myocardial fibrosis	Use	Y	Y	Y
10	630	M/W	65	21.2	196	7.0	ASCVD	566	Chronic undifferentiated schizophrenia; AAR	M/W	63	18.3	207	6.8	ASCVD	None	Y	Y	Y
		Mean	46.9	17.9	187.8	6.96					48.4	20.3	188.8	6.920		3Y/7N	8Y/2N	5Y/5N	5Y/5N
		SD	15.9	5.9	16.3	0.24					13.7	11.2	24.4	0.20					

[a] PMI, postmortem interval (hours); [b] Storage time (months) at -80C. Other abbreviations: ASCVD, arteriosclerotic cardiovascular disease; MCA, middle cerebral artery; ATOD, at time of death; ADC, alcohol dependence, current at time of death; ADR, alcohol dependence, in remission at time of death; AAC, alcohol abuse, current at time of death; AAR, alcohol abuse, in remission at time of death; ODC, other substance dependence, current at time of death; ODR, other substance dependence, in remission at time of death; OAC, other substance abuse, current at time of death; OAR, other substance abuse, in remission at time of death; VPA, valproic acid

bioRxiv preprint doi: https://doi.org/10.1101/2023.04.11.536217; this version posted April 11, 2023. The copyright holder for this preprint (which was not certified by peer review) is the author/funder, who has granted bioRxiv a license to display the preprint in perpetuity. It is made available under aCC-BY 4.0 International license.

Table S2.

**Variable**		**Factor**	***p*-value**
**Sum CB1R intensity**			
	**Paired analysis**	Main effect of subject group	$F_{(1,88)}$=7.532, p=0.007
		Main effect of cortical layer	$F_{(5,88)}$=4.803, p<0.001
	**Unpaired analysis**	Main effect of subject group	$F_{(1,91)}$=6.410, p=0.013
		Main effect of cortical layer	$F_{(5,91)}$=4.706, p<0.001
**Mean CB1R intensity**			
	**Paired analysis**	Main effect of terminal type	$F_{(2,280)}$=1053.740, p<0.001
		Subject group × terminal type interaction	$F_{(2,280)}$=34.872, p<0.001
		Post hoc	
		vGlut-IR effect	$F_{(1,280)}$=8.451, p=0.004
		high-CB1R-expressing vGAT-IR effect	$F_{(1,280)}$=69.393, p<0.001
	**Unpaired analysis**	Main effect of terminal type	$F_{(2,283)}$=1026.559, p<0.001
		Subject group × terminal type interaction	$F_{(2,283)}$=33.972, p<0.001
		Post hoc	
		vGlut-IR effect	$F_{(1,283)}$=8.575, p=0.004
		high-CB1R-expressing vGAT-IR effect	$F_{(1,283)}$=65.369, p<0.001

bioRxiv preprint doi: https://doi.org/10.1101/2023.04.11.536217; this version posted April 11, 2023. The copyright holder for this preprint (which was not certified by peer review) is the author/funder, who has granted bioRxiv a license to display the preprint in perpetuity. It is made available under aCC-BY 4.0 International license.

Table S3.

**Substance/ medication**	**Use history in SZ (n)**	**Mean of Ctrl/SZ sum CB1R intensity ratio (mean ± SEM)**	***p*-value**
**Cannabis**	**Yes (3)**	95.832 ± 9.026	t(8)=0.403
	**No (7)**	86.170 ± 4.726	*p*=0.697
**Antipsychotics**	**Yes (8)**	91.541 ± 4.783	t(8)=0.452
	**No (2)**	79.181 ± 9.326	*p*=0.664
**Antidepressants**	**Yes (5)**	95.131 ± 5.866	t(8)=0.557
	**No (5)**	83.006 ± 6.105	*p*=0.593
**Benzodiazepines/ valproic acid**	**Yes (5)**	105.216 ± 5.977	t(8)=1.699
	**No (5)**	72.910 ± 4.528	*p*=0.128

Abbreviations: Ctrl = unaffected comparison, SZ = schizophrenia, SEM = standard error of mean

bioRxiv preprint doi: https://doi.org/10.1101/2023.04.11.536217; this version posted April 11, 2023. The copyright holder for this preprint (which was not certified by peer review) is the author/funder, who has granted bioRxiv a license to display the preprint in perpetuity. It is made available under aCC-BY 4.0 International license.

Table S4.

Terminal type	Substance/ medication	Use history in SZ	Mean of Ctrl/SZ mean CB1R intensity ratio (mean ± SEM)	*p*-value
Excitatory (vGlut1-IR)	Cannabis	Yes	86.269 ± 20.197	t(8)=0.267
		No	80.159 ± 12.307	*p*=0.796
	Antipsychotics	Yes	81.696 ± 11.097	t(8)=-0.056
		No	83.174 ± 31.533	*p*=0.957
	Antidepressants	Yes	88.234 ± 14.661	t(8)=0.606
		No	75.750 ± 14.490	*p*=0.562
	BZD/VPA	Yes	97.432 ± 13.173	t(8)=1.712
		No	66.551 ± 12.315	*p*=0.125
High-CB1R-expressing Inhibitory (vGAT-IR)	Cannabis	Yes	140.101 ± 23.453	t(8)=1.104
		No	115.563 ± 10.910	*p*=0.302
	Antipsychotics	Yes	130.094 ± 10.448	t(8)=1.480
		No	92.246 ± 26.412	*p*=0.177
	Antidepressants	Yes	128.398 ± 15.658	t(8)=0.509
		No	117.451 ± 14.767	*p*=0.625
	BZD/VPA	Yes	126.176 ± 15.424	t(8)=0.299
		No	119.673 ± 15.330	*p*=0.773
Low-CB1R-expressing Inhibitory (vGAT-IR)	Cannabis	Yes	92.728 ± 7.645	t(8)=-0.456
		No	95.609 ± 2.757	*p*=0.660
	Antipsychotics	Yes	93.710 ± 3.391	t(8)=-0.730
		No	98.887 ± 0.375	*p*=0.486
	Antidepressants	Yes	95.181 ± 4.466	t(8)=0.149
		No	94.309 ± 3.784	*p*=0.885
	BZD/VPA	Yes	95.865 ± 4.620	t(8)=0.386
		No	93.625 ± 3.519	*p*=0.710

Abbreviations: BZD = benzodiazepine, Ctrl = unaffected comparison, SZ = schizophrenia, SEM = standard error of mean, VPA = valproic acid

bioRxiv preprint doi: https://doi.org/10.1101/2023.04.11.536217; this version posted April 11, 2023. The copyright holder for this preprint (which was not certified by peer review) is the author/funder, who has granted bioRxiv a license to display the preprint in perpetuity. It is made available under aCC-BY 4.0 International license.

Figure S1.

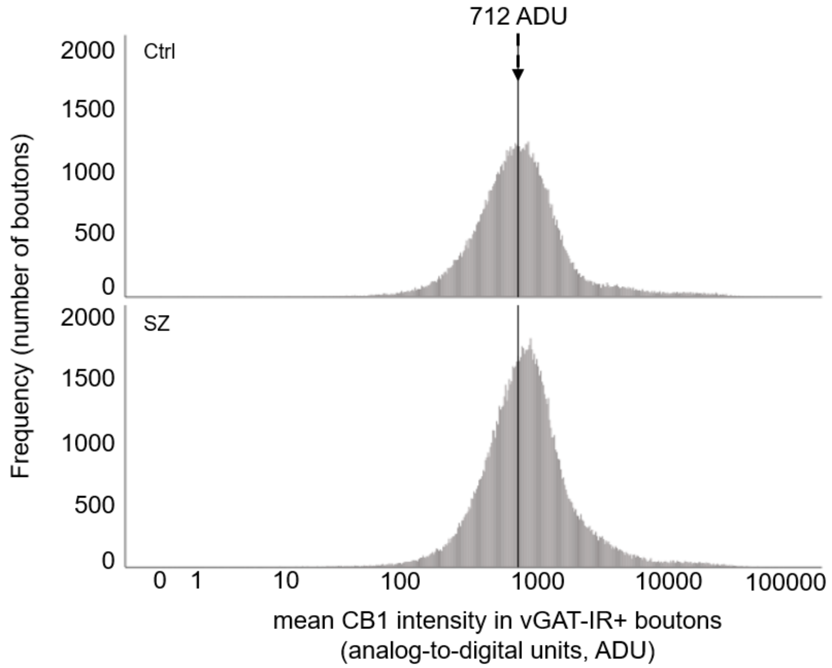

bioRxiv preprint doi: https://doi.org/10.1101/2023.04.11.536217; this version posted April 11, 2023. The copyright holder for this preprint (which was not certified by peer review) is the author/funder, who has granted bioRxiv a license to display the preprint in perpetuity. It is made available under aCC-BY 4.0 International license.

Figure S2.

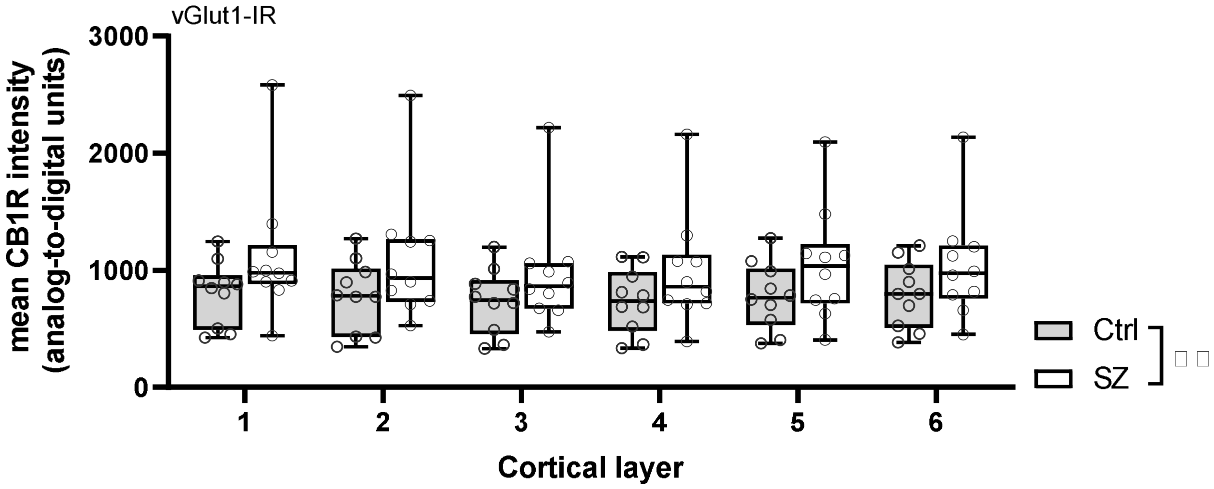

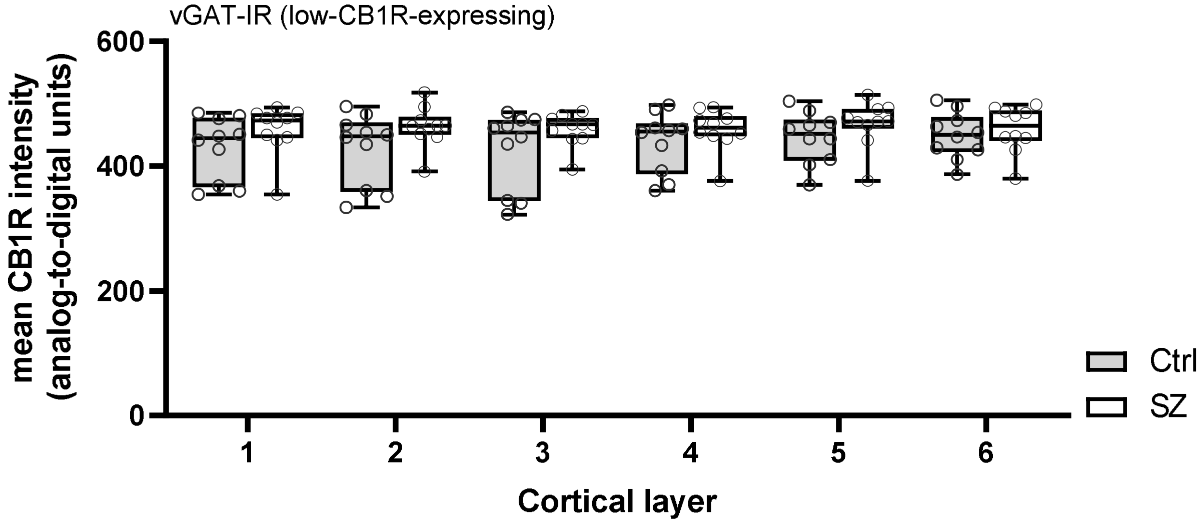

bioRxiv preprint doi: https://doi.org/10.1101/2023.04.11.536217; this version posted April 11, 2023. The copyright holder for this preprint (which was not certified by peer review) is the author/funder, who has granted bioRxiv a license to display the preprint in perpetuity. It is made available under aCC-BY 4.0 International license.

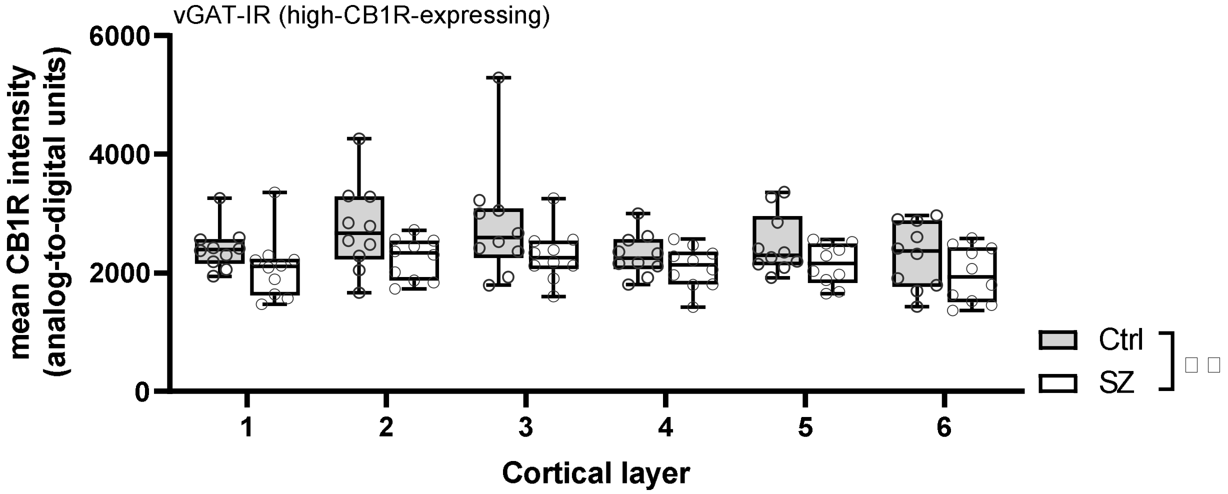

Supplement: Supplement 1 [file NIHPP2023.04.11.536217v1-supplement-1.pdf]
